# Enhancing drought forecasting with CNN-TQWT and metaheuristic hybrids: evidence from Norway

**DOI:** 10.1038/s41598-026-50063-7

**Published:** 2026-05-22

**Authors:** Sertaç Oruç, Türker Tuğrul, Mehmet Ali Hınıs

**Affiliations:** 1 The Center for Sámi Studies, UiT Norges Arktiske Universitet, N-9037 Tromsø, Norway; 2 Civil Engineering Department, Faculty of Engineering and Natural Sciences, Ankara Yıldırım Beyazıt University, Ankara, 06010 Türkiye; 3 Department of Civil Engineering, Faculty of Technology, Gazi University, Ankara, 06560 Türkiye; 4 Civil Engineering Department, Faculty of Engineering, Aksaray University, Aksaray, 68100 Türkiye

**Keywords:** Wavelet decomposition, SPI, TQWT, CNN, Drought forecasting, Machine learning, Climate sciences, Environmental sciences, Hydrology, Mathematics and computing

## Abstract

Droughts are among the most damaging natural hazards, exerting severe impacts on ecosystems, economies, and societies. While typically catastrophic, their effects can vary across regions, including polar areas where outcomes may differ unexpectedly. Accurate forecasting is therefore essential for climate adaptation and resource planning. This study analyzes monthly precipitation data from 1970 to 2025 across four Norwegian cities (Bergen, Kristiansand, Oslo, and Tromsø). Standardized Precipitation Index (SPI12) values were calculated and used as inputs to predictive models based on Convolutional Neural Networks (CNN). To enhance performance, the CNN framework was combined with Random Forest (RF) and also optimization techniques, Genetic Algorithm (GA), and Particle Swarm Optimization (PSO), as well as signal decomposition methods such as Variational Mode Decomposition (VMD) and the Tunable Q-Factor Wavelet Transform (TQWT). Four input configurations were tested, and results show that CNN–TQWT hybrid models consistently outperformed other approaches across all study sites, highlighting their potential for reliable drought prediction in diverse climatic settings. Specifically, the most effective models were identified as: Bergen: CNNTQWTM03 (R = 0.9732, NSE = 0.9231) Kristiansand: CNNTQWTM03 (R = 0.9879, NSE = 0.9568) Oslo: CNNTQWTM02 (R = 0.9727, NSE = 0.9172) Tromsø: CNNTQWTM01 (R = 0.9777, NSE = 0.9395). The findings highlight the superior accuracy of the TQWT decomposition method across all stations, underscoring its potential to support decision-makers in developing effective agricultural and water management policies. Furthermore, the findings of this study will contribute to the formulation of drought preparedness and development plans. By providing a technical foundation for proactive planning, these results will assist in mitigating the impacts of drought through more resilient management strategies.

## Introduction

Drought is a slow-onset hydroclimatic hazard with profound consequences for agriculture, water resources, and ecosystems. Driven primarily by human-induced climate change and rising global temperatures^1^, the impacts of drought are expanding globally. Projections show that beyond already arid regions like Africa, Northern and Central Europe will face significant drought risks^2–4^. Particularly in high-latitude areas, rising temperatures are altering the hydrological cycle; thus, even if rainfall increases, summer droughts are expected to recur with greater frequency and intensity^5^. The 2018 dry spell in Northern Europe underscored this vulnerability, demonstrating that abundant water resources offer no absolute immunity against the shifting climate^6,7^.

Consequently, assessing future drought scenarios is crucial for national adaptation and water resources strategies. Recent research has increasingly focused on predicting drought evolution using physically based projections and statistical or dynamical downscaling, and on translating these projections into SPI/SPEI-based drought scenarios^8, 9^. However, the specific hydro-climatic characteristics of regions with strong spatial gradients, such as mountainous and high-latitude environments, challenge the effectiveness of traditional statistical models and simple satellite-product evaluations, because rapid topographic changes, snow processes and mixed rain–snow regimes are difficult to represent consistently^10–13^. In such settings, data-driven and hybrid machine-learning approaches offer an attractive alternative for linking observed climate variables to drought indices at local scale^14–17^.

Recent work has started to move beyond traditional statistical and machine-learning models for SPI/SPEI forecasting towards deep learning; however, the coverage of architecture and hybridisation strategies remains fragmentary. A comprehensive review of deep learning models for drought prediction indicates that most operational and research applications are still dominated by single-model LSTM/GRU configurations and relatively shallow hybrids (e.g., wavelet–ANN, EMD–DBN). In contrast, CNN-based frameworks remain comparatively uncommon and are rarely evaluated systematically^14^.

Existing CNN studies demonstrate clear potential. For instance, Danandeh Mehr et al.^18^ developed a CNN–LSTM architecture for SPEI-3 and SPEI-6 forecasting at two stations in Ankara, showing that the hybrid model outperformed standalone CNN, standalone LSTM, and classical machine-learning benchmarks. Similarly, Chen et al.^19^ used a pure CNN to predict daily SAPEI at the basin scale, obtaining skilful 1–10-day forecasts by exploiting spatial information from gridded meteorological fields. Nevertheless, these studies typically examine a limited set of architectures (CNN, LSTM, CNN–LSTM) and do not explore how different hybridisation choices around a CNN backbone-such as meta-heuristic optimisation, ensemble coupling, or multi-scale decomposition—translate into gains in SPI-based drought prediction skill.

In parallel, there is a rapidly growing literature on decomposition- and optimisation-based hybrids for drought indices, yet these are largely decoupled from CNN-centred modelling. Decomposition methods such as the tunable Q-factor wavelet transform (TQWT) (Selesnick, 2011) and variational mode decomposition (VMD) have been successfully integrated with shallow learners and kernel methods. Latifoğlu and Özger^20^ showed that TQWT-based hybrids, particularly TQWT-GPR, substantially improve SPI3/SPI6 forecasts compared with standalone models and VMD/EMD-based configurations. Furthermore, Ekmekcioğlu^21^ demonstrated that a VMD-XGBoost model yields markedly higher NSE values than both standalone XGBoost and DWT-XGBoost for Palmer Drought Severity Index (PDSI) in a semi-arid Turkish basin. On the optimisation side, Mei et al.^22^ combined GRU and CNN in a single architecture where hyperparameters were tuned using a modified PSO algorithm, reporting significant performance gains over LASSO, RF, and a simple GRU model for predicting a monthly MCI drought index in Yunnan. Achite et al.^23^ utilized a range of machine learning methods, including Convolutional Neural Networks (CNN), to predict sediment parameters for the Bakhada and Sidi M’hamed Ben Aouda (SMBA) dams in Algeria. Their results indicated that the Deep Neural Network (DNN) outperformed the other models. Elbeltagi et al.^24^ developed a novel model for predicting local and global data by hybridizing CNN, RF, CNN-Extreme Gradient Boosting (XGB), and LSTM methods. They investigated the performance of forward-looking models using this hybrid approach, based on PDSI data calculated for the certain Egyptian stations. In their findings, they highlighted that the CNN-LSTM model yielded effective results. Nyamane et al.^25^ also investigated the performance of the CNN-LSTM hybrid model. They utilized different time scales of the SPEI for the Northern Cape region of South Africa and compared the performance of the CNN-LSTM model with the individual performances of ANN, LSTM, and CNN models. Their results indicated that the hybrid model outperformed the other models. Additionally, it is frequently emphasized by researchers in the literature that CNN is already a highly successful algorithm. It particularly stands out in the prediction of drought indices^26^. Tugrul et al.^27^ conducted a study on the forward-looking prediction of drought indices at the Apa Dam. In their research, they utilized machine learning techniques such as LSTM and SVM. To enhance model performance, various wavelet techniques were also employed. Their results emphasized that wavelet techniques significantly improved the overall performance of the models. This is shown in summary Table 1.

**Table 1:** Earlier literature studies.

Authors	Year	study area	Algorithms and methods used
Danandeh Mehr et al.^18^	2023	Ankara, Türkiye (SPEI-3/6 forecasting)	Hybrid CNN–LSTM, Standalone CNN, Standalone LSTM
Chen et al.^19^	2024	Fenhe River Basin, China (SAPEI prediction)	Pure CNN
Latifoğlu and Özger^20^	2023	SPI3/6 estimation	TQWT-GPR Hybrid, VMD, EMD
Ekmekcioğlu^21^	2023	Semi-arid basin, Türkiye (sc-PDSI forecasting)	VMD-XGBoost Hybrid, DWT-XGBoost
Mei et al.^22^	2022	Yunnan, China (MCI drought index)	Hybrid GRU-CNN, PSO (Optimization), LASSO, RF
Achite et al.^23^	2025	Algeria (Sediment parameter prediction)	CNN, DNN, and various Machine Learning methods
Elbeltagi et al.^24^	2024	Egyptian (PDSI)	CNN-LSTM, CNN, CNN-RF etc
Nyamane et al.^25^	2024	Northern Cape, South Africa (SPEI forecasting)	CNN-LSTM Hybrid, ANN, LSTM, CNN
Bora et al.^26^	2024	Drought prediction model	CNN
Tugrul et al.^27^	2025	Apa Dam (Drought index forecasting)	LSTM, SVM, and Wavelet techniques
Kachalla et al.^28^	2024	Electric Water Boiler Energy Prediction	ANN etc

In the literature, machine learning techniques are widely employed for the forward-looking prediction of various parameters. For instance, Kachalla et al.^28^ are among the researchers who leveraged machine learning techniques for Electric Water Boiler Energy Prediction. The prediction parameters in this field can be further diversified. Researchers frequently utilize both traditional methods and deep learning/machine learning techniques, particularly within the energy sector^29,31^.

Taking together, these studies indicate that both multi-scale decomposition and meta-heuristic optimisation can substantially enhance drought prediction. However, they have been applied mainly to tree-based models, shallow networks, or recurrent architecture rather than to CNN-based SPI/SPEI models. To our knowledge, there is still no systematic assessment of CNN, CNN-GA, CNN-RF, CNN-PSO, CNN-TQWT, and CNN-VMD configurations for SPI-driven drought forecasting, particularly in high-latitude hydroclimatic settings such as Norway. The present study is positioned to fill this methodological gap by bringing these CNN-centred hybrids into a unified comparative framework.

Scandinavia offers a distinctive experimental setting for drought research, combining cold, oceanic and subarctic regimes that differ markedly from arid or monsoon climates. Hydropower infrastructure, agricultural systems and fragile terrestrial and aquatic ecosystems in Norway are already showing increasing vulnerability to drought anomalies^32,33^. The country spans pronounced climatic contrasts: Oslo experiences relatively dry and warm summers, Kristiansand represents a transitional south-coast zone, Bergen is characterised by a very wet maritime regime, and Tromsø exhibits a high-latitude coastal–Arctic climate, with groundwater resources, hydropower production and local water supply being exposed in different ways across these settings^34,35^.

CNNs have demonstrated strong performance in the literature, particularly in noise filtering and feature extraction from time-series data. This study aims to augment their inherent capabilities. By incorporating optimization algorithms and wavelet decomposition, we sought to push the model’s performance boundaries, effectively resolving short-term signals and capturing complex temporal patterns. Therefore, CNN was the preferred machine learning algorithm in this study.

While previous work has examined SPI-based drought prediction in Norway using Random Forest and wavelet-supported LSTM architectures^16^, hybrid CNN-centred models across contrasting Norwegian climate regimes remain insufficiently studied. The main gap is not whether CNNs can be used for drought forecasting, but how forecast skill changes when the same SPI12 target is modeled under different hybridization strategies and different lagged input structures. In this study, we compare CNN, CNN-GA, RF, CNN-PSO, CNN-TQWT and CNN-VMD models for one-month-ahead SPI12 forecasting in Bergen, Kristiansand, Oslo and Tromsø^36,37^. By benchmarking these alternative frameworks against the standalone CNN, we evaluate gains in forecast accuracy, temporal stability and robustness across contrasting Norwegian climate settings, and we identify which model–lag combinations are most suitable for site-specific SPI12 forecasting^38–40^.

## Material and methods

**Fig. 1:**
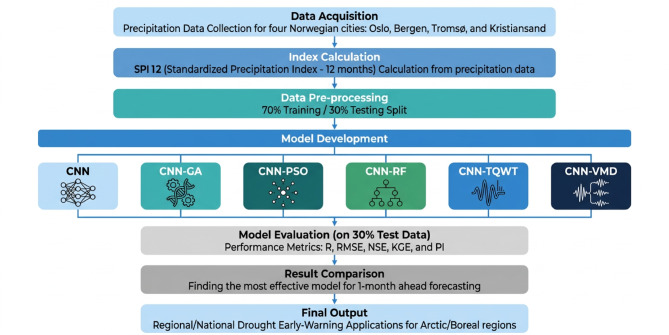
Workflow of SPI12-based one-month-ahead drought forecasting in Norway.

This section outlines the methodological framework used to develop and compare six models for one-month-ahead SPI12 forecasting in Norway: CNN, CNN-GA, RF, CNN-PSO, CNN-TQWT, and CNN-VMD **(**Fig. 1**)**. All models were trained using the same lagged SPI12 input structures and the same chronological training–testing partition for the four Norwegian study sites. The standalone CNN served as the deep-learning baseline. GA and PSO were used as optimization-based extensions of the CNN framework, whereas TQWT and VMD were used as signal-decomposition steps prior to CNN training. In addition, RF was trained on the same inputs as a benchmark tree-based learner. Accordingly, RF is interpreted here as a comparative baseline rather than as an unspecified post-processing stage applied to CNN outputs.

### Study area and dataset

The analysis focuses on six long-term meteorological stations that represent four study sites, Bergen, Kristiansand, Oslo-Blindern, Tromsø and the main hydro-climatic south-north Norway gradients. Bergen-Fredriksberg (SN50560) and Bergen-Florida (SN50540) on the hyper-maritime west coast, Mestad i Oddernes (SN39220) and Kjevik (SN39040) in the Kristiansand area on the south coast, and Oslo-Blindern (SN18700) in eastern Norway (Grøntoft, 2019) and Tromsø (SN90450). Together, these stations form a compact west–east and coastal–inland transect. The Bergen stations are located in steep coastal terrain exposed to moist North Atlantic westerlies; orographic uplift of onshore flow produces very high annual precipitation totals and frequent heavy-precipitation events, and rainfall can vary considerably over short distances in complex topography^41,42^. Local wind-shelter and spillover effects around the Bergen basin further modulate the near-surface wind field^43^, and Bergen’s humid maritime climate, with mild winters and precipitation on a large fraction of days, has led it to be described as Europe’s rainiest city^44^.

**Fig. 2:**
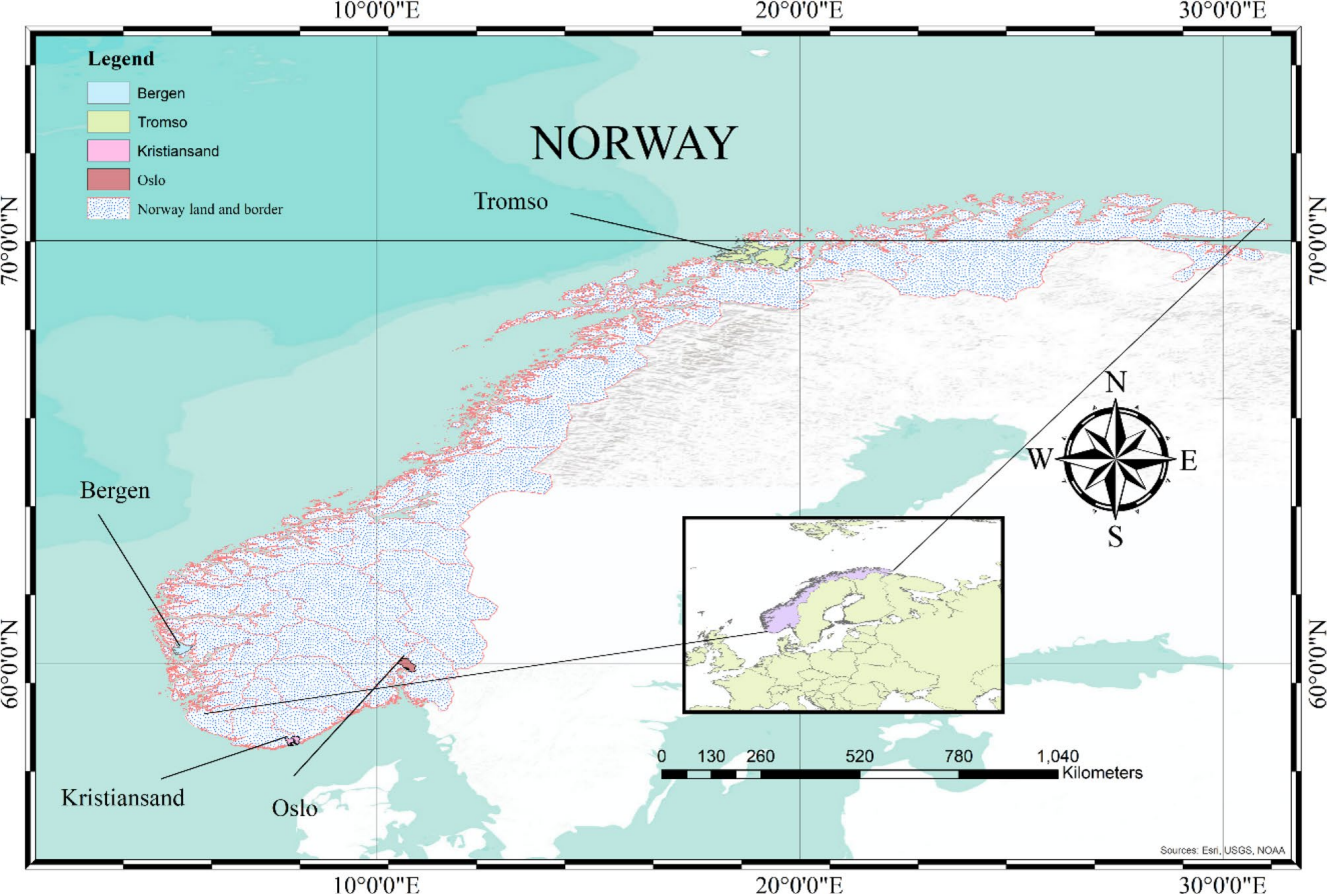
Study region (The map was generated using ArcMap (version 10.8) from ArcGIS Desktop; https://desktop.arcgis.com).

Farther south, Kjevik and Mestad Oddernes sample the milder south-coast climate, where annual precipitation and temperature conditions are intermediate between the very wet west coast and the drier inland east, while short-duration heavy rainfall events can be relatively intense along this coast^12^. Oslo-Blindern, situated east of the main orographic barrier, is representative of the more continental climate of southeastern Norway, with substantially lower annual precipitation totals and larger seasonal temperature contrasts than the Atlantic-facing west coast^45^. Moving north to Tromsø, the climate shifts to subarctic, where winters are longer and harsher, and summers are milder and shorter, shaping the seasonal dynamics of drought risk. Tromsø has reported temperature and precipitation changes during the last century, making the meteorological station representative of climate change in the region^46^. Taken together, these six stations (Table 2) provide a suitable basis for examining how meteorological drought characteristics derived from SPI12 vary between very wet maritime and more continental climate regimes in Norway (Fig. 2). Additional information about the station, based on their station ID, can be obtained from the Norwegian Climate Service Center “https://seklima.met.no”.

**Table 2:** Data statistics – precipitation.

Study site (station IDs)	start	end	mean (mm)	min (mm)	max (mm)	std. dev
Bergen (SN50560 and SN50540)	01–1970	10–2025	199.38	4.1	641.2	116.5
Kristiansand (SN39220 and SN39040)	01–1970	10–2025	145.9	0.0	612.5	95.0
Oslo (SN18700)	01–1970	10–2025	67.05	0.0	279.1	41.8
Tromsø (SN90450)	01–1970	10–2025	89.8	1.6	322.2	51.4

### Standardized precipitation index (SPI)

The SPI values, which can be calculated using the precipitation data measured at the stations, can also be calculated for gridded precipitation datasets (for example, satellite or radar precipitation data from remote sensing or meteorological model precipitation simulations). Owing to the widespread availability and computational ease of datasets, the WMO recommended the use of SPI as the meteorological drought index to monitor drought conditions. Because of the given use of ease and efficiency of calculating and evaluating drought characteristics, SPI was used in this study. SPI is calculated through utilizing the gamma distribution that is applied to the cumulative precipitation over a specific period of interest^47^:

1 $$G(x) = \frac{1}{{\beta^{\alpha } \Gamma (\alpha )}}\int_{0}^{x} {x^{\alpha - 1} } e^{{ - \frac{x}{\beta }}} dx$$

where G(x) is the CDF of the gamma distribution, α and β are the shape and scale parameters that are estimated with the cumulative precipitation for which it is not zero.

Then, SPI values can be estimated through the inverse CDF of the standard normal distribution:

2 $$SPI = F_{n}^{ - 1} [G(x)]$$

where $F_{n}^{ - 1} (x) = \frac{1}{{\sqrt {2\pi } }}\int_{ - \infty }^{x} {e^{{ - \frac{{x^{2} }}{2}}} }$ is the inverse CDF of the standard normal distribution.

Positive SPI values represent precipitation greater than the mean value (wet period) and negative SPI values represent drier periods. Since SPI is normalized, wet and dry climates can be expressed in a similar manner. The recommended threshold values for SPI commonly used in the literature are developed by McKee et al.^47^; 2.0 and above indicate extremely wet conditions, while values between 1.50 and 1.99 represent severely wet conditions, and values from 1.00 to 1.49 denote moderately wet conditions. Near normal conditions are characterized by SPI values ranging from -0.99 to 0.99. On the dry end of the spectrum, moderate drought is indicated by SPI values between -1.00 and -1.49, severe drought by values from -1.50 to -1.99, and extreme drought by values of -2.0 and below.

Depending on the length of the accumulation period (i.e., between 1 and 48), SPI reflects different types of droughts. Longer accumulation periods reflect slowly accumulating dry conditions in which the adverse impacts last longer, whereas shorter accumulation periods indicate dry periods that may occur and disappear relatively in shorter time frames. For example, SPI-12 is widely used as a proxy indicator for medium- to long-term drought impacts, while SPI1 reflects meteorological drought.

### Model structure

**Fig. 3:**
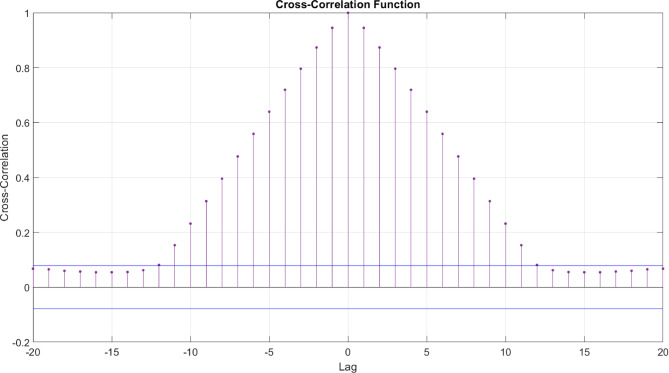
Results of cross-correlation based on SPI12.

Model input structure is one of the parameters that strongly affects drought-forecast performance. In this study, candidate lagged inputs were defined from the cross-correlation screening and then organized as four nested SPI12 input structures (M01–M04). This design was used to test whether progressively longer persistence information improved one-month-ahead forecasting skill. Figure 3 summarizes the lag-window screening used to define the candidate structures, and Table 3 lists the final lag combinations. Cross-correlation was selected because it provides a transparent and reproducible first-pass screening of lag dependence in hydrological time series^17,27,48–51^. At the same time, the selected lag sets should be interpreted as literature-supported candidate structures rather than universally optimal input orders; alternative feature-selection methods such as mutual information, partial correlation, and MRMR remain worthwhile for future work.

**Table 3:** Model input structures.

model	SPI12 based on lagged time												output
M01	t-9	t-8	t-7	t-6	t-5	t-4	t-3	t-2	t-1				t
M02	t-10	t-9	t-8	t-7	t-6	t-5	t-4	t-3	t-2	t-1			t
M03	t-11	t-10	t-9	t-8	t-7	t-6	t-5	t-4	t-3	t-2	t-1		t
M04	t-12	t-11	t-10	t-9	t-8	t-7	t-6	t-5	t-4	t-3	t-2	t-1	t

### Convolutional neural network (CNN) model

Convolutional neural networks (CNNs) are a family of deep learning architectures. Exploiting local connectivity and shared weights allows them to extract informative local patterns from the input while keeping the number of trainable parameters relatively low^52–54^. This makes CNNs particularly attractive for learning spatial and temporal structures in hydro-climatic data, especially when gridded fields or lagged sequences of predictors are used as inputs^18,19,39^.

A CNN typically consists of a sequence of convolutional layers, optionally followed by pooling layers and one or more fully connected layers. Through successive convolutional operations, the network learns hierarchical feature representations directly from the raw input: small filters slide over the data and respond to local structures—such as edges and textures in images, or short-term anomalies in a time series—which are then combined in deeper layers into more abstract, task-relevant features^52,53^. Recent hydrological applications, for example CNN–LSTM–RF integrations for discharge prediction, further illustrate how this learned feature hierarchies can be embedded within hybrid architectures to improve forecasting skill^55^.

A typical CNN combines three main layer types: convolutional, pooling and fully connected layers^56^. Convolutional layers apply multiple filters across the input to produce feature maps, i.e. transformed representations that highlight relevant patterns. Pooling layers then downsample these feature maps, reducing their dimensionality while retaining the most informative responses. Finally, one or more fully connected layers aggregate the extracted features and map them to the desired output, such as a class label or a continuous prediction. The discrete convolution of an input *X* with a filter *K* can be written as^52^:

$$S(i,j)=\sum_{m}\sum_{n}X(i+m,j+n)K(m,n)$$

where *S(i,j)* denotes the value of the output feature map at position *(i,j)* and the summation runs over the filter dimensions.

Because of their local connectivity and weight sharing, CNNs capture spatial dependencies in the data in a computationally efficient way and use far fewer parameters than fully connected networks. This has made them highly effective for tasks such as image recognition, object detection and related visual applications^57,58^. At the same time, their ability to learn task-relevant features automatically, without explicit manual feature engineering, is one of the main reasons why CNNs have become a standard tool in modern machine learning.

### Random forest (RF)

Random forest (RF), introduced by Breiman^59^, is a machine learning technique used for both the classification and the regression problems^60^. By utilizing a single dataset, multiple models are trained, which also increases the prediction accuracy. In practice, the method follows three stages as follows: data subdivision, obtaining decision trees for these subdivisions, and development of the final prediction by averaging the predictions obtained from trees^60,61^. Random forest has several key advantages, such as high accuracy-low errors and computational speed as Choi et al.^62^ indicated. Moreover, ease of hyper-parameter tuning during runtime is also an advantage for RF compared to ANN and SVR^60,63,64^. For more details, readers can refer to Breiman^59^, Biau and Scornet^64^, Yu et al. ^65^, and Tyralis et al.^63^.

### Genetic algorithm (GA)

Genetic Algorithms (GAs), first introduced by Holland in the 1960s, and 1970s apply Darwinian principles of evolution to computational optimization by evolving populations of candidate solutions through selection, crossover, and mutation^66^. Their gradient-free search capability makes them effective for both continuous and discrete problems, and they have been successfully applied in diverse domains such as parameter optimization, scheduling, linguistic analysis, and fuzzy network tuning^67–72^.

The algorithm proceeds through three core stages. These are; initializing a random population, applying genetic operators through probabilistic rather than deterministic process, and evaluating solution quality until convergence criteria trigger termination^72,73^. When solutions fail to meet established quality thresholds, the algorithm iterates through another generational cycle, systematically refining and enhancing the population through selection pressure and genetic variation, that enables the algorithm to be apllied for different problem domains^72,74^.

These steps are:

1. Crossover (stochastic): part of two solutions “is swapped” to produce new ones.
2. Mutation (stochastic): part of a new solution “is flipped” to generate a new one and prevent it from converging into local optima.
3. Selection: the new solutions are evaluated according to the objective function, and the best candidates are selected.

In certain instances, such as a high mutation rate that could lead to the loss of good solutions, the elitism operator is employed to guarantee that the optimal solutions are transferred to the next generation without modification, ensuring that the best candidates are maintained within the solution set^71,74^.

### Particle swarm optimization (PSO)

Kennedy and Eberhart introduced particle swarm optimization (PSO) in 1995, drawing conceptual inspiration from coordinated and collective animal behaviors observed in natural systems such as schools of fish navigating currents and bird flocks foraging collectively. Unlike genetic algorithms and other comparable evolutionary methods, PSO achieves more rapid convergence while requiring reduced computational resources, particularly when applied to nonlinear optimization problems^75,76^. The algorithm treats each potential solution as a “particle” moving through an n-dimensional space, where n corresponds to the number of problem’s parameters^77^.

Initially, particles are positioned randomly across the solution space. As the algorithm progresses through successive iterations, each particle adjusts its trajectory based on two guiding influences: the particle’s best position discovered so far, and the globally best position identified by any particle of the entire swarm. This dual-component memory system drives particles toward potential solution regions while simultaneously maintaining exploration capability^78–82^. The search continues until particles converge on a shared optimal location, signaling that the algorithm has identified the best available solution accessible within the search space^83–85^.

### Tunable Q-factor wavelet transform decomposition (TQWT)

Wavelet-based decompositions are widely used in hydroclimatic time-series analysis because they provide joint time–frequency localization and allow nonstationary signals to be represented at multiple characteristic scales^86–88^. In this study, the tunable Q-factor wavelet transform (TQWT)^89^ was employed as a preprocessing step to break down drought-related time series into quasi-stationary sub-components prior to model training.

Unlike the conventional Discrete Wavelet Transform (DWT), the TQWT is a redundant transform (oversampled transform) that permits perfect reconstruction while offering adjustable redundancy through its tunable Q-factor^89^. By varying the Q-factor, oscillatory and transient features of the signal can be effectively separated, enabling more precise characterization of underlying phenomena^90,91^. For further details, readers may consult Selesnick^89^ and Yang and Wang^91^.

### Variational mode decomposition (VMD)

Variational Mode Decomposition (VMD), by Dragomiretskiy and Zosso^92^, is a non-recursive, fully adaptive signal decomposition method designed to extract intrinsic modes of a time series based on their spectral characteristics and to overcome some limitations of the traditional decomposition methods. Unlike traditional empirical approaches such as EMD, VMD formulates the mode decomposition task as a constrained variational optimization problem Dragomiretskiy and Zosso (2014), aiming to minimize the bandwidth of each mode around its central frequency^93^, demonstrating higher efficiency in removing high frequency noises without diminishing much of the signal amplitude^18,94^.

VMD initially transforms the real-valued mode ${u}_{k}$ into an analytic signal ${u}_{k}^{+}$ with a single-sided frequency spectrum:

3 $${u}_{k}^{+}\left(t\right) = \left(\delta \left(t\right) + \frac{j}{\pi t}\right)\times {u}_{k}(t)$$

A given sample *X* is decomposed into k eigenmode components (IMF) with the same centre frequency while ensuring that the sum of the estimated bandwidth of each IMF is the smallest. The corresponding constraint variational expression is defined as follows^92^:

4 $$\underset{{u}_{k}{\omega}_{k}}{\mathrm{min}}\left\{\sum_{k=1}^{K}\Vert {\partial}_{t}\left[{u}_{k}^{+}(t){e}^{-j{\omega}_{k}t}\right]\Vert \genfrac{}{}{0pt}{}{2}{2}\right\}, \mathrm{s}\mathrm{u}\mathrm{b}\mathrm{j}\mathrm{e}\mathrm{c}\mathrm{t} \mathrm{t}\mathrm{o} \sum_{k=1}^{K}{u}_{k}\left(t\right) = f(t)$$

The limitation in the aforementioned equation can be mitigated by using a quadratic penalty and Lagrangian multipliers *λ (t)*. The augmented Lagrangian is defined as follows:

5 $$\mathcal{L}\left(\left\{{u}_{k}\right\},\left\{{\omega}_{k}\right\},\lambda \right) = a\sum_{k=1}^{K}\Vert {\partial}_{t}\left[{u}_{k}^{+}(t){e}^{-j{\omega}_{k}t}\right]\Vert \genfrac{}{}{0pt}{}{2}{2} + f\left(t\right) - \sum_{k=1}^{K}{u}_{k}\left(t\right)\Vert \genfrac{}{}{0pt}{}{2}{2} + \langle \lambda \left(t\right),f\left(t\right) - \sum_{k=1}^{K}{u}_{k}\left(t\right)\rangle$$

where α denotes the balancing parameter of the “data-fidelity” constraint.

VMD addresses several shortcomings, including pattern mixing, endpoint effects, and the challenge of defining precise stopping conditions compared to other techniques^92,95^. Accordingly, these features make it useful for analyzing complex, non-stationary signals in fields including hydrology and structural health monitoring^57,92,95,96^.

#### Performance metrics

Various statistical metrics were used for performance assessment. These metrics are the correlation coefficient (R), the root mean square error (RMSE), the Nash–Sutcliffe efficiency (NSE), Kling–Gupta efficiency (KGE) and Performance Index (PI), defined in Eqs. (6), (7), (8), (9), (10), respectively. When these parameters, NSE, R, and KGE, approach 1, and PI and RMSE and MAE approach 0, it indicates that the model estimations are good. In Eqs. (6) - (10), ${x}_{pi}$  = the predicted value, ${x}_{oi}$  = the observed value, N = the number of data, $\overline{{x }_{o}}$  = average observed value, and $\overline{{x }_{p}}$ = average predicted value.

Correlation coefficient (*r*) can be calculated as in Eq. (6)^48^:

6 $$r = \frac{\sum_{i=1}^{N}{(x}_{pi}-\overline{{x }_{p}})\left({x}_{oi}-\overline{{x }_{o}}\right)}{\sqrt{\sum_{i=1}^{N}{{(x}_{pi}-\overline{{x }_{p}})}^{2}}*\sqrt{\sum_{i=1}^{N}{\left({x}_{oi}-\overline{{x }_{o}}\right)}^{2}}}$$

RMSE is calculated in Eq. (7)^51^:

7 $$RMSE = \sqrt{\frac{1}{N}{\sum}_{i=1}^{N}{\left({x}_{oi}-{x}_{pi}\right)}^{2}}$$

NSE is calculated in Eq. (8)^49,97^:

8 $$NSE = 1-\left[\frac{\sum_{i=1}^{N}{\left({x}_{oi}-{x}_{pi}\right)}^{2}}{\sum_{i=1}^{N}{\left({x}_{oi}-\overline{{x }_{o}}\right)}^{2}}\right]$$

Also, Kling–Gupta efficiency (KGE)^98,99^ and Performance Index (PI) performances criteria were used to determine model performances (Eqs. (9) and (10)):

$$KGE = 1 - \sqrt{{\left(r-1\right)}^{2} +{ \left(a-1\right)}^{2} + {\left(\beta -1\right)}^{2}}$$

9 $$\beta = \frac{{x}_{p}}{{x}_{o}}, a = \frac{{\sigma}_{{x}_{p}}}{{\sigma}_{{x}_{o}}}$$

10 $$PI =\frac{\frac{RMSE}{\left|\overline{{x }_{p}}\right|}}{1+\mathrm{r}}$$

11 $$\mathrm{MAE}=\mathrm{N}1 i=1\sum\mathrm{N} \vert\mathrm{xo,i} -\mathrm{xp,i} \vert$$

## Results

In analyses conducted with machine learning techniques, model performances are influenced by the data’s training and testing ratios. For model evaluation, the data were partitioned chronologically, with the earlier 70% used for training and the later 30% used for testing, so that temporal order was preserved. This design allows a consistent comparison across all model variants, but the reported metrics should still be interpreted as hold-out performance rather than as the most exhaustive possible time-series validation. Within this common evaluation framework^48–51^ the hybrid CNN configurations were compared across four lagged input structures for each study site. This study hybridizes several optimization techniques and wavelet decomposition methods with the CNN deep learning method and compares their analytical performances. Using data obtained from six meteorological stations that represent four study sites, Bergen, Kristiansand, Oslo–Blindern, Tromsø in Norway, SPI12 values were initially calculated, and the results were analyzed using machine learning techniques with four different model input structures. The results of these analyses are presented in Table 4. The most effective performance among all methods used for the Bergen was achieved by the CNNTQWTM03 model (R = 0.9732, NSE = 0.9231, KGE = 0.7073, PI = 0.4348, and RMSE = 0.2766). Furthermore, considering the developed model input structures, M03 outperformed the other model structures. This indicates that lagged SPI12 values must be kept at an optimum level when creating the model input structure and that the input structure of M03 should be utilized. Specifically, for this location, in analyses conducted with optimization techniques, CNN-PSO/M01 and CNN-GA/M04 emerged as competitive configurations, both clearly trailed by the TQWT-based variants. In anayses performed without any optimization techniques, CNNM01 exhibited more effective performance compared to other model input structures (M02, M03, and M04). Although performance metrics close to CNNPSOM01 were obtained, optimization techniques (GA, PSO) positively impacted the model results. Another important finding obtained for this station is that decomposition methods are more effective than optimization techniques.

**Table 4:** Results of all models.

Bergen																		
CNN							CNN-GA						CNN-RF					
Model	R	NSE	KGE	PI	RMSE	MAE	R	NSE	KGE	PI	RMSE	MAE	R	NSE	KGE	PI	RMSE	MAE
**M01**	**0.9041**	**0.8036**	**0.6217**	**0.7201**	**0.4421**	**0.2847 **	0.9058	0.7940	0.5612	0.7368	0.4528	0.2988	0.8390	0.6730	0.8383	0.9620	0.5704	0.3601
M02	0.8946	0.7841	0.6033	0.7587	0.4634	0.2946	0.8771	0.6540	0.0655	0.9694	0.5867	0.3948	0.8103	0.6357	0.5735	1.0535	0.6152	0.3761
M03	0.8984	0.7953	0.6329	0.7373	0.4513	0.2931	0.9053	0.7682	0.4890	0.7817	0.4802	0.3182	**0.8549**	**0.7023**	**0.8053**	**0.9100**	**0.5442**	**0.3372 **
M04	0.8957	0.7969	0.7327	0.7354	0.4495	0.2836	**0.8983**	**0.8063**	**0.8661**	**0.7172**	**0.4389**	**0.2795 **	0.8112	0.5910	0.7095	1.0924	0.6379	0.3971
CNN-PSO							CNN-TQWT						CNN-VMD					
Model	R	NSE	KGE	PI	RMSE	MAE	R	NSE	KGE	PI	RMSE	MAE	R	NSE	KGE	PI	RMSE	MAE
**M01**	**0.8982**	**0.8037**	**0.7757**	**0.7220**	**0.4419**	**0.2840 **	0.9723	0.9084	0.4940	0.4747	0.3018	0.2005	**0.9586**	**0.9154**	**0.8897**	**0.4594**	**0.2901**	**0.1923 **
M02	0.8634	0.6293	0.3222	1.0108	0.6073	0.3961	0.9714	0.9050	0.5300	0.4837	0.3074	0.2002	0.9530	0.8982	0.7701	0.5055	0.3183	0.2122
M03	0.9037	0.7740	0.4571	0.7725	0.4742	0.3075	**0.9732**	**0.9231**	**0.7073**	**0.4348**	**0.2766**	**0.1761 **	0.9439	0.8890	0.8687	0.5302	0.3323	0.2131
M04	0.8928	0.7873	0.6757	0.7537	0.4600	0.2981	0.9514	0.8850	0.5100	0.4637	0.2874	0.1961	0.9452	0.8891	0.8266	0.5297	0.3322	0.2142
Kristiansand																		
CNN							CNN-GA						CNN-RF					
Model	R	NSE	KGE	PI	RMSE	MAE	R	NSE	KGE	PI	RMSE	MAE	R	NSE	KGE	PI	RMSE	MAE
M01	0.9324	0.8380	0.6209	0.5537	0.4015	0.3275	0.9425	0.8321	0.5490	0.5607	0.4087	0.3485	0.8732	0.7402	0.7047	0.7233	0.5084	0.4328
**M02**	**0.9381**	**0.8508**	**0.6122**	**0.5298**	**0.3853**	**0.3173 **	0.9413	0.8687	0.6986	0.4961	0.3614	0.2927	0.8866	0.7649	0.6594	0.6831	0.4836	0.3984
M03	0.9348	0.8418	0.5782	0.5463	0.3967	0.3228	**0.9377**	**0.8742**	**0.8019**	**0.4865**	**0.3537**	**0.2832 **	**0.9110**	**0.8117**	**0.8811**	**0.6035**	**0.4328**	**0.3579 **
M04	0.9352	0.8435	0.5712	0.5433	0.3945	0.3247	0.9369	0.8646	0.7012	0.5049	0.3670	0.2972	0.8416	0.6556	0.4389	0.8470	0.5854	0.4750
CNN-PSO							CNN-TQWT						CNN-VMD					
Model	R	NSE	KGE	PI	RMSE	MAE	R	NSE	KGE	PI	RMSE	MAE	R	NSE	KGE	PI	RMSE	MAE
M01	0.9368	0.8550	0.6363	0.5225	0.3798	0.3121	0.9801	0.9428	0.7665	0.3210	0.2385	0.1977	0.9649	0.8949	0.6847	0.4385	0.3233	0.2739
**M02**	**0.9409**	**0.8634**	**0.6920**	**0.5061**	**0.3686**	**0.2984 **	0.9845	0.9491	0.7678	0.3022	0.2250	0.1877	0.9518	0.8836	0.7862	0.4645	0.3402	0.2723
M03	0.8581	0.5037	0.0159	1.0078	0.7027	0.5901	**0.9879**	**0.9568**	**0.7673**	**0.2778**	**0.2072**	**0.1770 **	0.9628	0.9035	0.7444	0.4206	0.3098	0.2554
M04	0.9076	0.7156	0.4209	0.7431	0.5319	0.4627	0.9360	0.8082	0.6669	0.6013	0.4369	0.3185	**0.9671**	**0.9058**	**0.7402**	**0.4147**	**0.3061**	**0.2522 **
Oslo																		
CNN							CNN-GA						CNN-RF					
Model	R	NSE	KGE	PI	RMSE	MAE	R	NSE	KGE	PI	RMSE	MAE	R	NSE	KGE	PI	RMSE	MAE
**M01**	**0.9038**	**0.7858**	**0.6702**	**0.4044**	**0.4616**	**0.3089 **	0.9124	0.8019	0.6784	0.3872	0.4440	0.3017	**0.8384**	**0.6708**	**0.7391**	**0.5192**	**0.5723**	**0.3862**
M02	0.8943	0.7657	0.6519	0.4251	0.4828	0.3249	0.9058	0.7968	0.7055	0.3935	0.4496	0.3040	0.7701	0.5555	0.7264	0.6266	0.6650	0.4662
M03	0.8972	0.7626	0.6238	0.4272	0.4859	0.3279	**0.9043**	**0.8111**	**0.8011**	**0.3796**	**0.4335**	**0.2925 **	0.8169	0.6462	0.7762	0.5446	0.5933	0.4063
M04	0.8670	0.7108	0.6018	0.4792	0.5364	0.3653	0.8994	0.6433	0.3356	0.5231	0.5957	0.4269	0.7935	0.5326	0.6715	0.6341	0.6819	0.4685
CNN-PSO							CNN-TQWT						CNN-VMD					
Model	R	NSE	KGE	PI	RMSE	MAE	R	NSE	KGE	PI	RMSE	MAE	R	NSE	KGE	PI	RMSE	MAE
M01	0.8607	0.5488	0.2906	0.6006	0.6700	0.4629	0.9665	0.9098	0.7698	0.2541	0.2996	0.2038	**0.9464**	**0.8717**	**0.7777**	**0.3061**	**0.3572**	**0.2310**
M02	0.8044	0.3272	0.0756	0.7562	0.8181	0.5729	**0.9727**	**0.9172**	**0.7668**	**0.2426**	**0.2869**	**0.1981 **	0.9427	0.8517	0.7286	0.3297	0.3841	0.2506
M03	**0.8982**	**0.7716**	**0.6505**	**0.4188**	**0.4767**	**0.3210 **	0.9650	0.9028	0.7739	0.2639	0.3109	0.2129	0.9251	0.8277	0.7339	0.3587	0.4140	0.2676
M04	0.8103	0.6357	0.5735	1.5359	0.6152	0.3892	0.9606	0.8811	0.7181	0.2926	0.3439	0.2350	0.9064	0.8163	0.8073	0.3741	0.4275	0.2911
Tromso																		
CNN							CNN-GA						CNN-RF					
Model	R	NSE	KGE	PI	RMSE	MAE	R	NSE	KGE	PI	RMSE	MAE	R	NSE	KGE	PI	RMSE	MAE
**M01**	**0.8980**	**0.7952**	**0.6275**	**1.1214**	**0.4514**	**0.3590 **	0.8127	0.6326	0.3346	1.5726	0.6046	0.4259	**0.8550**	**0.7203**	**0.7143**	**1.3408**	**0.5275**	**0.4023 **
M02	0.8937	0.7860	0.6010	1.1487	0.4614	0.3672	0.8054	0.6349	0.5156	1.5738	0.6026	0.4314	0.8456	0.6969	0.6621	1.4029	0.5491	0.4229
M03	0.8940	0.7868	0.5946	1.1465	0.4606	0.3632	**0.9051**	**0.8014**	**0.5506**	**1.1002**	**0.4445**	**0.3496 **	0.8203	0.6557	0.6035	1.5159	0.5852	0.4432
M04	0.8824	0.7590	0.5455	1.2264	0.4896	0.3811	0.8865	0.6805	0.2396	1.4090	0.5638	0.4320	0.8238	0.6436	0.5160	1.5393	0.5954	0.4548
CNN-PSO							CNN-TQWT						CNN-VMD					
Model	R	NSE	KGE	PI	RMSE	MAE	R	NSE	KGE	PI	RMSE	MAE	R	NSE	KGE	PI	RMSE	MAE
M01	0.8981	0.7751	0.3645	1.1749	0.4730	0.3734	**0.9777**	**0.9395**	**0.6954**	**0.5848**	**0.2453**	**0.1840 **	0.9521	0.8694	0.5379	0.8706	0.3605	0.2594
M02	0.9003	0.7557	0.1129	1.2233	0.4930	0.3893	0.9725	0.9253	0.7298	0.6515	0.2726	0.1950	**0.9573**	**0.8897**	**0.5723**	**0.7978**	**0.3312**	**0.2466 **
**M03**	**0.8982**	**0.7865**	**0.5421**	**1.1449**	**0.4609**	**0.3611 **	0.9678	0.8959	0.7060	0.7712	0.3219	0.2400	0.9545	0.8760	0.4969	0.8473	0.3512	0.2570
M04	0.8699	0.6570	0.0272	1.4729	0.5841	0.4588	0.9720	0.9068	0.6343	0.7278	0.3044	0.2250	0.9517	0.8677	0.5144	0.8766	0.3629	0.2695

The most successful models in their group are shown in bold.

When the results for Kristiansand, another station constituting the study area, are examined, it is observed that, similar to the Bergen station, the CNNTQWTM03 model demonstrated the most superior performance among all methods (R = 0.9879, NSE = 0.9568, KGE = 0.7673, PI = 0.2778, and RMSE = 0.2072). At this station as well, the results obtained with wavelet decomposition methods successfully outperformed the optimization techniques. Although the most successful method and model input structure show similarities with Bergen (such as CNNTQWTM03), like Bergen (where CNNPSOM01 was superior), one of the good results among the optimization techniques was obtained with CNNGAM03. Another finding for this station is that the performance metrics were generally found to be more effective compared to the Bergen station. The performance metrics of almost all models are more efficient than those of Bergen. In fact, the most effective performance metrics across all methods and models used in the entire analysis were obtained at this specific station.

Oslo is another station located in the southern region of Norway where meteorological measurements are conducted. When the results obtained from this station are examined in general, the performance metrics obtained from the analyses conducted solely with CNN, without any optimization techniques, surpassed those of the CNNRF and CNNPSO techniques. In other words, in some models, optimization methods negatively affected the model performance metrics. Apart from these, the most successful model among the methods used at this station was CNNTQWTM02 (R = 0.9727, NSE = 0.9172, KGE = 0.7668, PI = 0.2426, and RMSE = 0.2869). CNNVMDM01 follows this model in terms of performance, as the non-TQWT alternative. The TQWT method is remarkably successful compared to the other methods utilized in this study.

In the analyses conducted for Tromsø, the most effective results among the methods used were again obtained with CNNTQWT. When this model was analyzed with the M01 input structure (CNNTQWTM01), performance metrics of R = 0.9777, NSE = 0.9395, KGE = 0.6954, PI = 0.5848, and RMSE = 0.2453 were achieved. Consequently, throughout this study, TQWT yielded more effective results across all stations compared to the other preferred methods. Another notable finding from this station is that performance metrics were generally negatively affected in analyses conducted with RF and GA. As for GA, it only outperformed the performance metrics obtained from standalone CNN in the M03 model input structure; however, in other model input structures, it fell behind the performance of the standalone CNN analyses.

**Fig. 4:**
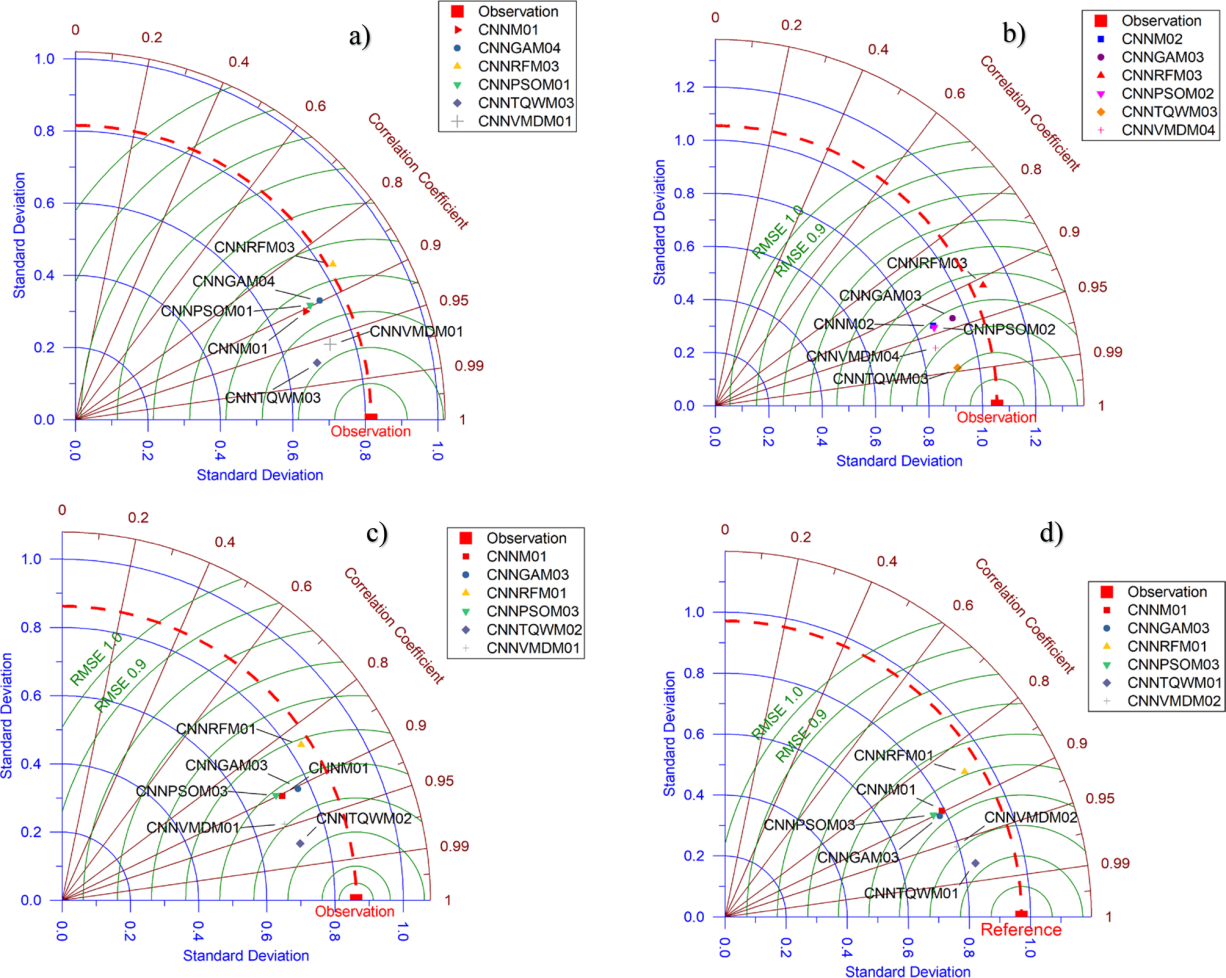
Taylor Diagrams for the most successful models in their respective groups: **a**) Bergen **b**) Kristiansand **c**) Oslo **d**) Tromsø.

Figure 4 presents the Taylor diagram to compare the most successful models in their respective groups with other models. Upon examining this diagram, the most effective model among the methods used for Bergen is CNNTQWTM03. Compared to the other models in the diagram, it is the closest to the observed values. This demonstrates the success of each algorithm and model. Looking at the other stations, the CNNTQWTM03 model for Kristiansand, the CNNTQWTM02 model for Oslo, and the CNNTQWTM01 model for Tromsø have been more effective than the other methods used in this study. It is evident from this figure that the CNNTQWT algorithm is generally successful. Furthermore, identical results were confirmed through the examination of performance metrics.

**Fig. 5:**
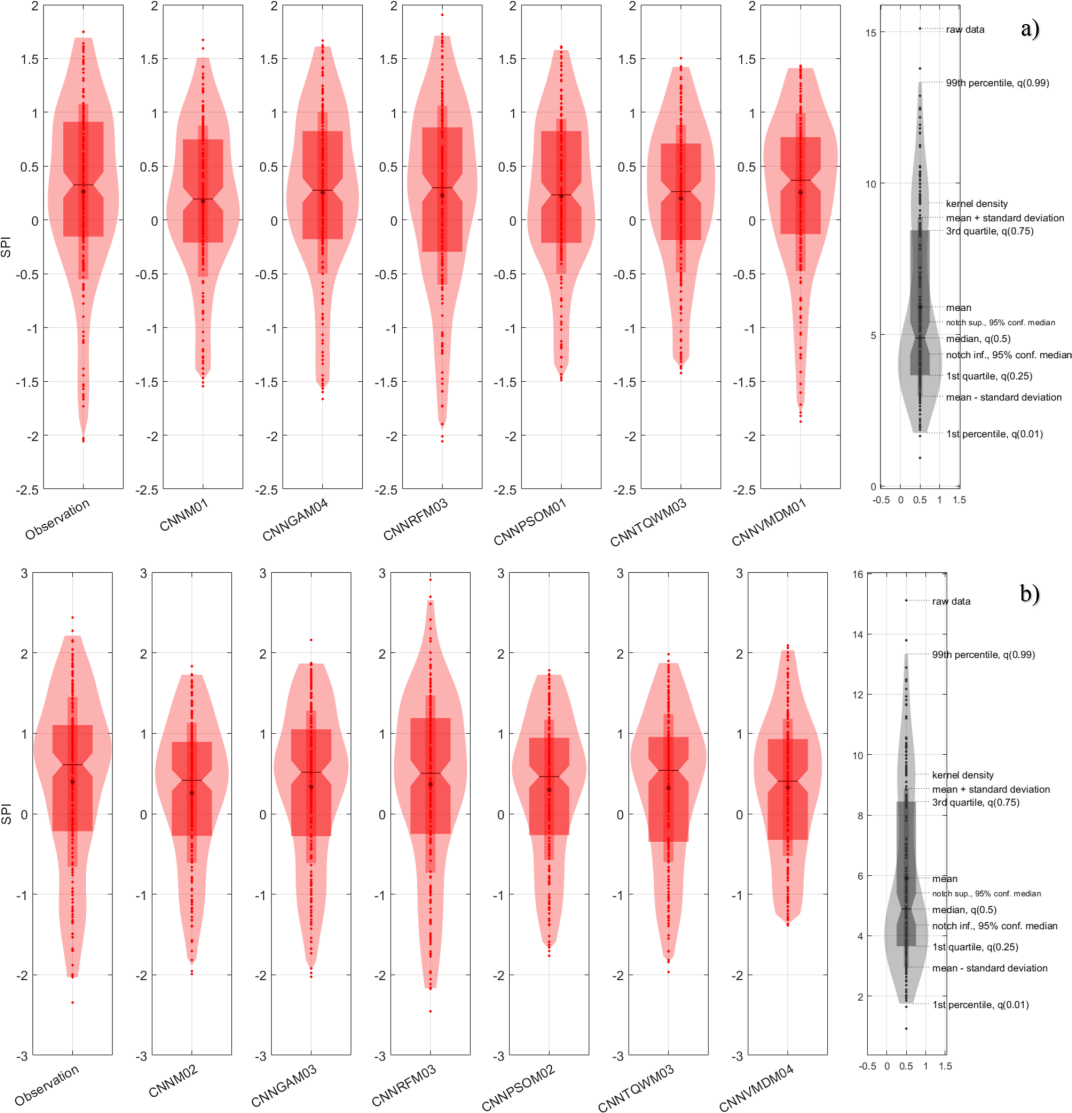

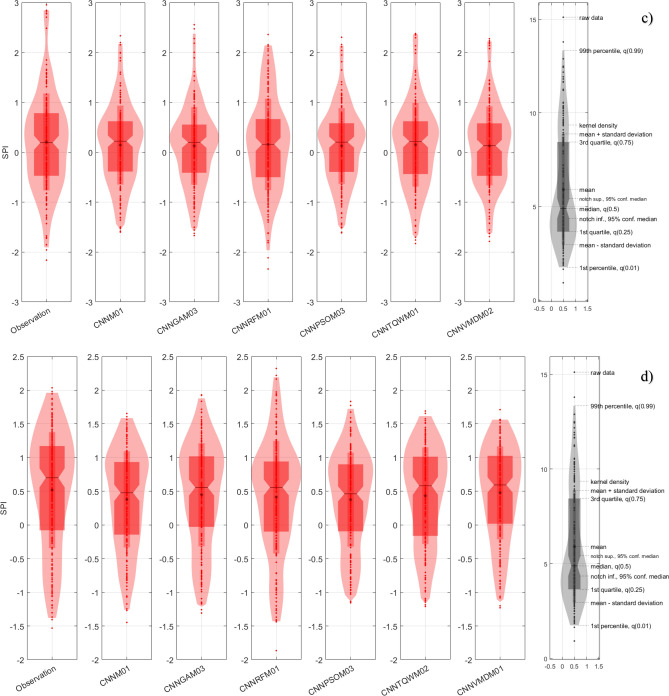
Violin Diagrams for the most successful models in their respective groups: **a**) Bergen **b**) Kristiansand **c**) Oslo **d**) Tromsø.

Similar to the Taylor Diagram, Fig. 5 also used to illustrate the most successful models within their respective groups using Violin plots for each specific station. The Violin plot compares observed values and predictive models based on their statistical properties, such as mean, median, first quartile, third quartile, and kernel density. In addition to statistical characteristics, it also reveals structural match. When examining the results for the Bergen station, the CNNVMDM01 model appears to exhibit morphological consistency. Despite this morphological alignment, however, examination of statistical properties reveals significant differences in kernel densities. Specifically, the median value within the 95% confidence interval notably deviates from the observed value. Consequently, morphological similarity alone is insufficient to designate this model as the most successful compared to other methods. Considering the median and mean values, measures of central tendency, CNNTQWTM03 aligns most effectively with the observed values, making it more successful than the other methods in this category. Violin plot representations were generated for all stations. Analysis of the graph for Kristiansand shows that CNNTQWTM03 is the model that most closely resembles the observed values both statistically and visually. The features that most distinguish this model from other predictive models are its mean, kernel density, and median values. Regarding the visuals for Oslo and Tromsø, when mean and median values are considered, the most successful models are CNNTQWTM02 and CNNTQWTM01, respectively. All results obtained from the Violin plots are consistent with the results derived from the performance metrics.

**Fig. 6:**
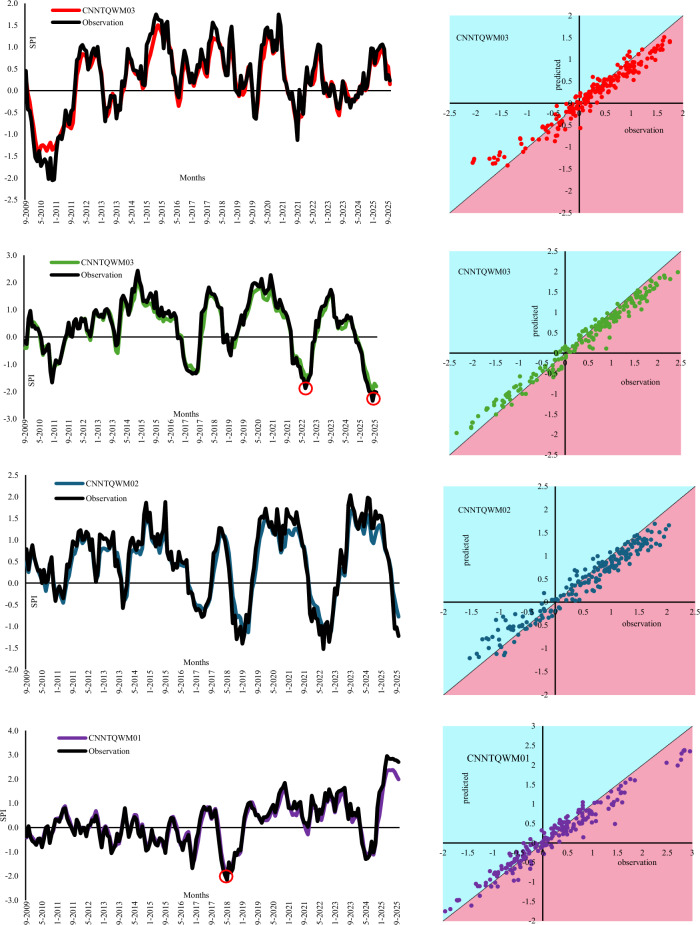
Time-series representation and scatter plots of the best-performing models in their respective groups: **a**) Bergen **b**) Kristiansand **c**) Oslo **d**) Tromsø.

Figure 6 illustrates the time-series comparison between the observed values and the best-performing models identified for each station. Additionally, scatter plots for the predictive models and observed values of these stations are presented in this figure. As shown, the prediction model for the Bergen station failed to sufficiently estimate the peak values in the observed data. In the scatter diagram, the predicted values do not align closely with a 1:1 straight line. Similarly, for the other stations, peak values could not be estimated with sufficient efficacy, consistent with the findings at the Bergen station. However, the results for the Kristiansand station demonstrate that the observed and predicted values were estimated more effectively compared to the other stations. Upon examining the scatter plot for this station, the identified values exhibit a more linear distribution compared to the results from the other stations.

Although the best-performing CNN-TQWT models reproduce the overall temporal evolution of SPI-12 with high fidelity, Fig. 6 shows a consistent attenuation of the most negative peaks. In particular, observed months approaching or exceeding the severe drought threshold (SPI-12 <= -1.5) are often predicted at less negative values, and months near the extreme drought threshold (SPI-12 <= -2.0) are only sparsely represented in the test period. This pattern is consistent with the rarity of such events and with the fact that overall regression training is dominated by the much larger number of near-normal and moderately dry months. Consequently, the models are more reliable for tracking general drought tendency than for reproducing the exact magnitude of the rarest drought extremes.

## Discussion

In this study, SPI12 values were first calculated using monthly total precipitation data obtained from four study sites, Bergen, Kristiansand, Oslo, Tromsø, in Norway; subsequently, these values were analyzed using the CNN deep learning method within four different model input structures. To enhance model performance, wavelet decomposition and optimization methods were hybridized with the CNN approach. The results generally indicate that wavelet decomposition methods and optimization techniques strengthened the model outcomes. However, in certain models and stations, utilizing the CNN without hybridization proved to be more effective than using optimization techniques. Such findings are commonly encountered in the existing literature^17^. Apart from these, performance metrics improved significantly in the results obtained using wavelet decomposition methods. In particular, numerous studies in the literature indicate that the (VMD method is frequently utilized in drought prediction, and many even provide performance comparisons between VMD and Empirical Mode Decomposition (EMD)^21,48,100–102^. Researchers have reported that VMD generally yields relatively better results compared to EMD. In fact, this is one of the primary reasons for selecting VMD in this study. Furthermore, the TQWT method has recently emerged as a novel approach utilized by researchers in wavelet decomposition studies. This study aimed to determine which method—among both optimization and wavelet decomposition techniques—most effectively improves model performance. It was found that, depending on the model input structure, the TQWT method achieved highly effective improvements across all stations when compared to all other methods. The efficacy of this method has also been highlighted by several researchers in the literature^89,103^.

The integration of TQWT and VMD with CNNs introduces a series of methodological advantages that substantially enhance the quality and interpretability of data prior to deep learning. Both decomposition techniques enrich the time–frequency domain by separating the raw signal into structured, denoised, and physically meaningful subcomponents. This preprocessing step ensures that the CNN does not operate on complex or mixed-frequency raw inputs, but rather on representations that isolate dominant oscillatory behaviors, amplitude–frequency modulations, and low to high scale dynamics. As a result, the learning process becomes more stable, more efficient, and more aligned with the intrinsic structure of hydro-meteorological and climatic signals.

In addition, TQWT and VMD address one of the fundamental challenges in environmental and engineering signal analysis: non stationarity. Since climatic, hydrological, and geophysical time series exhibit strong temporal variability, conventional CNNs struggle to extract consistent patterns from raw inputs. TQWT and VMD mitigate this limitation by capturing time localized oscillations and adaptive frequency content, which allows the CNN to learn features that are robust to seasonal shifts, sudden transitions, or multi scale variability. TQWT, with its tunable quality factor (Q), offers explicit control over oscillatory persistence, enabling the decomposition to align with harmonic, periodic, or quasi-stationary behaviors frequently observed in natural processes. VMD, by contrast, introduces variational adaptivity and decomposes signals into narrow band AM–FM intrinsic mode functions with data driven center frequencies, which is highly effective for signals with drifting spectral characteristics.

Furthermore, the complementary nature of TQWT and VMD enhances the multi-scale learning capabilities of CNNs. TQWT generates redundant, overcomplete representations that preserve oscillatory structures across scales, while VMD provides compact, mathematically constrained components that highlight dominant spectral modes. When these decomposition outputs are fused as CNN inputs, the network gains access to a richer feature space capturing both stable oscillatory patterns and transient modulations. This expanded representational diversity reduces feature ambiguity, improves separability among physical processes, and enhances generalization, particularly when forecasting or modeling complex environmental variables. In turn, the hybrid frameworks consistently outperform standalone CNN models by achieving higher accuracy metrics such as R^2^ and lower error measures including RMSE and MAE.

Finally, empirical findings from this study indicate that TQWT demonstrates notable superiority over VMD in the context of climatic and hydrological oscillations where periodicity, harmonic structure, and resonance phenomena are dominant. The tunable Q-factor enables TQWT to align its sub bands with the intrinsic oscillatory modes of such signals more precisely than VMD’s AM–FM decomposition. Although VMD remains highly effective for non-stationary spectral variations and frequency-modulating behaviors, TQWT’s capacity to produce sparsely distributed, stable sub bands makes it particularly well suited for modeling oscillatory hydrological processes. Therefore, the combined decomposition -CNN framework not only enhances prediction accuracy but also strengthens the physical interpretability and methodological robustness of the modeling pipeline, offering a powerful approach for hydrological and climate data analysis.

Cross-domain studies also support this finding; Zeng et al.^104^ directly combines TQWT and VMD for real world PCG (Phonocardiogram) signals and reports comparative performance. They found that TQWT provides more stable, oscillation-controlled features, while VMD gives adaptive refinement. On real PCG data, TQWT produces cleaner initial features than VMD alone, and combining them outperforms VMD by itself. Their results show that TQWT provides stable, interpretable sub band structure and VMD fine tunes AM–FM content but is sensitive to its parameters (K/α). Yuan et al.^105^ investigated a real-world rotating machinery study and showed that VMD parameters (K, α) were difficult to optimize and significantly affect performance. They emphasized that after optimized VMD, TQWT + sparse code shrinkage was applied. They concluded that TQWT sharpened impulses, improved sparsity, and enhanced periodic transient signatures.

A limitation shared by all model families is the underestimation of severe and extreme drought peaks. In this setting, the learned representations are dominated by the central part of the SPI-12 distribution, whereas the rarest negative excursions contribute relatively little to the overall optimization objective. Consequently, even the strongest decomposition-based models tend to smooth the lowest observed values toward less severe predictions. Addressing this issue would likely require either event-weighted loss functions, targeted augmentation or oversampling of severe months, or explicit threshold-based post-processing. These extensions were beyond the scope of the current comparative study but represent an important priority for future work.

Although the performance of optimization methods lagged behind that of wavelet decomposition methods, they have yielded quite satisfactory results in literature studies. In this research, while effective outcomes were obtained through optimization techniques, the performance improvements were not as substantial as those achieved with wavelet decompositions. Consequently, the latter techniques tend to overshadow the optimization methods. PSO and GA are among the techniques that have proven themselves in many fields, not just in hydrology. In this study as well, results close to those of wavelet decomposition methods were obtained in certain regions and models^106^.

A review of the literature reveals that the use of neural network-based approaches in hydrological modeling is becoming increasingly common. These models are frequently compared by researchers against widely preferred methods such as LSTM and MamGA to evaluate their performance. Furthermore, models like the Improved Arctic Puffin Optimization (IAPO) algorithm are being adopted to predict streamflow using various optimization techniques. It is recommended that researchers explore these methods in future studies^107,108^.

Finally, the input parameters for models M01 and M04 differ. This is directly related to the model input or the state of the data received. GA performance may vary depending on the search space characteristics and the landscape of the objective function. The adopted hyperparameters and candidate ranges for the investigated models are summarized in Table 5.

**Table 5:** Hyper parameters.

Category	Component / Model	Hyperparameter	Suggested value / Range
CNN architecture	Base Layers	Number of Filters	32
		Kernel Size	5
		Activation Function	ReLU
		Pooling Layer	2
		Dropout Rate	0.2
Training setting	All Models	Optimizer	Adam
		Learning Rate	0.001
		Batch Size	32
		Epochs	200
Meta-heuristic optimization	CNN-GA	Population Size	40
		Generations	80
		Crossover Probability	0.8
		Mutation Probability	0.1
	CNN-PSO	Number of Particles	35
		Max Iterations	100
		Acceleration Coefficients (c1, c2)	2.0, 2.0
		Inertia Weight (w)	0.9
Ensemble learning	CNN-RF	Number of Trees (n_estimators)	100, 200, 500
		Maximum Depth	15
		Min Samples Split	3
Signal decomposition	CNN-TQWT	Q-factor	3
		Redundancy (r)	3
		Decomposition Levels (J)	3
	CNN-VMD	Balancing Parameter (Alpha)	2000
		Time Step (Tau)	0
		Number of Modes (K)	3

### Limitations

This study has several limitations. First, the analysis is restricted to four Norwegian study sites represented by six long-term precipitation stations, which limits the spatial generalizability of the findings.

Second, only four nested lag-based input structures were evaluated, and alternative feature-selection methods such as mutual information, MRMR, or wrapper-based approaches were not tested. Third, model assessment relied on a chronological 70/30 train–test split; although this preserves temporal order, rolling-origin evaluation would provide a stronger test of time-series generalization.

Finally, drought characterization was based on SPI-12 derived from precipitation alone. SPI-12 is a precipitation-based meteorological drought indicator and a proxy for medium- to long-term drought impacts rather than a standalone hydrological drought index. In addition, the analysis focused mainly on overall regression metrics (R, NSE, KGE, RMSE, and MAE). As Fig. 6 indicates, strong aggregate performance does not automatically imply reliable detection of severe (SPI-12 <= -1.5) and extreme (SPI-12 <= -2.0) drought months. Future studies should therefore include explicit event-based categorical metrics, such as POD, FAR, CSI, and HSS, and examine rare-event-aware training strategies. 

## Conclusion

In this study, results obtained by hybridizing the CNN deep learning method with specific optimization and wavelet decomposition techniques are presented using four different model input structures. The major findings are highlighted as follows:

- Across the experiments, decomposition-based hybrids produced the most consistent improvements, and CNN-TQWT was the strongest overall performer.
- The best-performing site-specific configurations were CNNTQWTM03 for Bergen, CNNTQWTM03 for Kristiansand, CNNTQWTM02 for Oslo, and CNNTQWTM01 for Tromsø.
- The optimal lag structure was not uniform across sites, showing that regional hydroclimatic differences materially affect model skill and that input design should be location-specific.
- Optimization-based hybrids did not consistently improve on the standalone CNN; in Oslo, GA- and PSO-based variants often reduced performance relative to the baseline.
- These results indicate that signal decomposition can add more value than optimization-based hybridization for SPI12 forecasting in the Norwegian settings examined here.

At the same time, strong overall regression performance should not be interpreted as definitive proof of skill for the most severe drought events, which remain harder to reproduce.

Operationally, site-specific SPI12 forecasts may support staged drought preparedness discussions for reservoir operation, irrigation planning, and regional water-resources management when predicted conditions move toward severe or extreme drought classes.

Future work should extend the station network, test alternative lag-selection methods and validation schemes, and include explicit severe/extreme-event metrics to determine how robust these conclusions remain under stricter forecasting assessments.

## Data Availability

The original contributions presented in the study are included in the article. The raw data supporting the conclusions of this article will be made available by the authors upon reasonable request.
